# Identification of candidate gene for the defective kernel phenotype using bulked segregant RNA and exome capture sequencing methods in wheat

**DOI:** 10.3389/fpls.2023.1173861

**Published:** 2023-06-05

**Authors:** Hao Tang, Huixue Dong, Xiaojiang Guo, Mengping Cheng, Maolian Li, Qian Chen, Zhongwei Yuan, Zhien Pu, Jirui Wang

**Affiliations:** ^1^ State Key Laboratory of Crop Gene Exploration and Utilization in Southwest China, Sichuan Agricultural University, Chengdu, China; ^2^ Ministry of Education Key Laboratory for Crop Genetic Resources and Improvement in Southwest China, Sichuan Agricultural University, Chengdu, China

**Keywords:** BSE-seq, BSR-seq, AK58, exome capture, *HMGS-7A*

## Abstract

Wheat is a significant source of protein and starch worldwide. The defective kernel (Dek) mutant *AK-3537*, displaying a large hollow area in the endosperm and shrunken grain, was obtained through ethyl methane sulfonate (EMS) treatment of the wheat cultivar Aikang 58 (AK58). The mode of inheritance of the *AK-3537* grain Dek phenotype was determined to be recessive with a specific statistical significance level. We used bulked segregant RNA-seq (BSR-seq), BSA-based exome capture sequencing (BSE-seq), and the ΔSNP-index algorithm to identify candidate regions for the grain Dek phenotype. Two major candidate regions, DCR1 (Dek candidate region 1) and DCR2, were identified on chromosome 7A between 279.98 and 287.93 Mb and 565.34 and 568.59 Mb, respectively. Based on transcriptome analysis and previous reports, we designed KASP genotyping assays based on SNP variations in the candidate regions and speculated that the candidate gene is *TraesCS7A03G0625900* (*HMGS-7A*), which encodes a 3-hydroxy-3-methylglutaryl-CoA synthase. One SNP variation located at position 1,049 in the coding sequence (G>A) causes an amino acid change from Gly to Asp. The research suggests that functional changes in *HMGS-7A* may affect the expression of key enzyme genes involved in wheat starch syntheses, such as *GBSSII* and *SSIIIa*.

## Introduction

Wheat (*Triticum aestivum* L.) is a major global crop, providing approximately 20% of the total caloric intake for the world’s population (Ogbonnaya et al., 2013; Ma et al., 2022). Therefore, maintaining high grain quality and yield of wheat is essential for food security (Shiferaw et al., 2013). Wheat grain, the reproductive and storage organ, plays a crucial role in wheat propagation, spread, and yield and primarily consists of the embryo, endosperm, and seed coat (Ebrahimnejad and Rameeh, 2016). In wheat, grain filling refers to the process of starch biosynthesis and accumulation in the endosperm (Ahmed et al., 2015). Starch is the main component of wheat grain, accounting for 65%–70% of dry grain weight (Housley et al., 1981), and it significantly impacts wheat flour quality (Mancebo et al., 2015).

Starch synthesis in wheat grain starts after fertilization and continues until approximately 35 days, when the grain matures and dries (Khatun and Ahmed, 2015). When starch is synthesized in the endosperm, sucrose produces through leaf photosynthesis and enters the cytoplasm, serving as the carbon source for starch synthesis in wheat (Guo et al., 2015). Multiple factors influence wheat grain development, including wheat tissue organs (Barneix, 2007), starch synthesis-related enzymes, plant hormones, and environmental factors. Relevant tissue organs include leaves and stem sheaths. At the same time, starch synthesis-related enzymes encompass sucrose synthase (SuSy), ADP-glucose pyrophosphorylase (AGPase), granule-bound starch synthase (GBSS), starch synthase (SS), starch branching enzyme (SBE), starch debranching enzyme (DEB), starch phosphorylase (SP), and sucrose convertase (SC), among others (Wang et al., 2014). Plant hormones affecting wheat grain development include ethylene (ET), brassinosteroid (BR), gibberellin (GA), and abscisic acid (ABA), among others (Liu et al., 2013; Xiong et al., 2022). Environmental factors such as temperature, light, and soil moisture also play a role (Liu et al., 2016; Jiang et al., 2017; Djanaguiraman et al., 2020; Mirosavljević et al., 2021).

Defective kernel (Dek) mutants exhibit shrunken grains, and grain filling in crop Dek mutants is drastically impaired (Wang et al., 2017). Generally, the Dek phenotype reduces grain weight and significantly affects grain appearance and seed vigor (Li et al., 2017; Fu et al., 2019; Qi et al., 2019). Many Dek mutants have been identified in maize and rice, and numerous genetic loci regulating grain fullness have also been discovered. Dek mutants, such as *Dek10*, *Dek35*, *Dek36*, *Dek3*7, *Dek39*, *Dek40*, and *Dek42* (Chen et al., 2017; Qi et al., 2017; Wang et al., 2017; Dai et al., 2018; Li et al., 2018; Ren et al., 2019; Zuo et al., 2019), display germinated mutant kernels that are lethal during the seedling stage; *Dek15*, *Dek38*, *Dek41*, and *Dek44* (Lid et al., 2002; Garcia et al., 2017; He et al., 2019; Qi et al., 2019; Zhu et al., 2019) seeds cannot germinate at all, resulting in lethal embryo mutations. For example, the *Dek15* gene encodes sister chromatid cohesion protein 4 (SCC4), and mutation of this gene disrupts the cell cycle and nuclear replication, leading to the complete failure of seed germination (He et al., 2019). The *Dek38* gene encodes TEL2-interaction protein 2 (TTI2) molecular chaperone protein, which affects the development of male germ cells (Garcia et al., 2017). The *Dek1* gene, located in the 47.1 to 47.4 Mb region on chromosome 1 in maize, is involved in the differentiation and development of maize aleurone cells. A mutation of this gene leads to embryo lethality and affects the development of the aleurone layer and the accumulation of endosperm gliadin content (Lid et al., 2002; Song et al., 2020). At present, only a few Dek-related studies have been reported in Triticeae. Three QTLs, *QDek.Caas-3BS.1*, *QDek.Caas-3BS.2*, and *QDek.Caas-4AL*, associated with wheat grain Dek were identified using wheat mutant groups, explaining 14.78%–18.17%, 16.61%–21.83%, and 19.08%–28.19% of phenotypic variances, respectively (Fu et al., 2019). The loss-of-function mutation of the *sex6* (*SSIIa*) gene on chromosome 7H in barley causes amylopectin synthesis to decrease to less than 20% of the wild-type level. Simultaneously, the mutation also affects the binding of starch synthetases SSI, SBEIIa, and SBEIIb to starch granules and ultimately causes barley grain to become shrunken (Dek) (Morell et al., 2003). The *barley’s sex6 (SSIIa) mutant* was crossed with the *amo1* (*SSIIIa*) mutant to generate the *sex6amo1* double mutant, which produces high-amylose starch. The level of granule-bound starch synthase I (GBSSI) protein in starch granules increased, and starch synthase I (SSI), SSIIa, starch branching enzyme IIa (SBEIIa), and SBEIIb also significantly increased in the starch granules. The double mutant’s Dek phenotype was restored to a normal grain phenotype, indicating that changes in starch synthase function in cereal crops can also lead to shrunken grains (Li et al., 2011). These genes are crucial for synthesizing and accumulating starch and protein in the endosperm. Therefore, excavating DeK-related genes will be conducive to improving crop grain yield and quality.

This study analyzed Aikang58 (AK58) and its Dek mutant line *AK-3537* to investigate grain characteristics in different environments over several years. *AK-3537* exhibited poor grain filling, collapsed abdominal grooves, and shrunken grains. Transcriptome analysis using RNA-seq was performed on the grains of AK58 and *AK-3537*, and an F_2_ population with 130 individuals was constructed using *AK-3537* and AK58. Two high-confidence candidate regions, DCR1 (Dek candidate region 1) and DCR2, regulating wheat grain Dek, were mapped using BSE-seq (Bulked Segregant Exome Capture Sequencing) and BSR-seq (Bulked Segregant RNA-seq). We identified *TraesCS7A03G0625900* in the DCR1 region as a candidate gene for the wheat grain Dek phenotype using KASP (Kompetitive Allele-Specific Polymerase Chain Reaction) markers, the Chinese Spring reference genome (RefSeq 2.1), and the AK58 genome. This study will contribute to a deeper understanding of the regulatory mechanisms underlying wheat grain morphology and provide new insights to improve wheat yield through breeding.

## Materials and methods

### Plant materials

The EMS (ethyl methane sulfonate) mutant Dek line *AK-3537* originated from the wheat variety AK58. The Dek phenotype was stably inherited after eight generations of self-pollination. For genetic analysis and mapping, *AK-3537* was crossed with AK58 to produce an F_2_ population of 130 individuals. Wheat materials were cultivated in experimental fields and greenhouses at Sichuan Agricultural University, Chengdu Chongzhou (103° 38’ E, 30° 32’ N), Wenjiang (103° 51’ E, 30° 43’ N), and Xishuangbanna (99° 56’ E, 21° 08’ N), China. All field trials were well irrigated and managed following local standard practices, and all *AK-3537* × AK58 F_2_ plants were grown in a greenhouse at 20°C with a 16-h/8-h light/dark cycle.

In the field, a total of 20 plants from AK58 (10 individuals) and *AK-3537* (10 individuals) were randomly selected to investigate agronomic traits, including plant height (PH), tiller number (TN), heading date (HD), flag leaf length (FLL), and flag leaf width (FLW), using the method reported in previous studies. Thousand kernel weight (TKW), grain length (GL), and grain width (GW) were also measured using previously reported methods (Liu et al., 2017). Excel 2019 (Microsoft, Redmond, WA, USA) was used to calculate the phenotypic data. Analysis of variance was conducted, and individuals were ranked through Duncan’s test and plotted using GraphPad Prism V9.0.0. R software (version 4.2.1) was used as a plotting tool to calculate the wheat Dek phenotypic data.

### Identification of Dek phenotype

After maturity, wheat grains were harvested and threshed by hand. One hundred grains were randomly sampled from each individual, and the sampling was repeated three times. The wheat grains were visually examined for the Dek phenotype, and the incidence (percentage of grain Dek phenotype) was calculated. To better observe the wheat grain Dek phenotype, the grains of AK58 and *AK-3537* were stored at 4°C in FAA (Formalin-Aceto-Alcohol) fixative (ensuring the kernels did not float on the surface of the fixative solution) during the wheat grain filling stage. Subsequently, the cell structures of the normal phenotype and Dek phenotype were observed using frozen and free-hand sections (Leica CM1860) and x-ray computed tomography (Micro-CT).

### The BSE-seq and BSR-seq for rapid map Dek gene

(*AK-3537* × AK58) F_1_ grains were observed at the mature grain period in the natural field. F_2_ populations were used for genetic analysis. We then performed a chi-square test (χ^2^) to test phenotypic data (grain Dek phenotype) for a goodness of fit to the ratio of 3:1 expected for a single gene (or semi-dominant) genetic basis in Excel 2019 by CHISQ.TEST function (*p* > 0.05 means no deviation from expectations of 3:1). Using the combined approaches of BSE-seq and BSR-seq, AK58, *AK-3537*, and the F_2_ population (grains with normal phenotype and Dek phenotype, with a mix of 30 random individuals each) were selected for DNA and RNA segregant pools. Leaves were collected for DNA extraction using the Plant Genomics DNA Extraction Kit (BIOFIT^®^, DN32-100, Chengdu, China), and grains (10–20 days post-anthesis, DPA) were sampled to extract total RNA using the Plant RNA Extraction Kit (BIOFIT^®^, RN34050, Chengdu, China). RNA sequencing generating 150 bp paired-end reads was performed on the Illumina HiSeq™ × platform. Clean RNA-seq data were mapped onto the Chinese Spring reference genome (RefSeq 2.1) and the AK58 genome (Jia J et al., 2021) using the software Bowtie2, and SNP calling was performed with the SAMtools software. The newly designed exome capture probe panel (Dong et al., 2020) and ΔSNP-index algorithm (Abe et al., 2012) were used to map the grain Dek gene in wheat rapidly. High-quality reads were aligned to the Chinese Spring reference genome (RefSeq 2.1) and the AK58 genome (Jia J et al., 2021) with default parameters. The parental AK58 sequencing data (DNA and RNA sequencing data) were used as a “background” to identify the causal mutation based on the assumption. Calculations were analyzed on PlantGmap (Zhang et al., 2021) (http://183.223.252.63:3333/).

Lastly, considering the characteristics of EMS mutagenesis, certain variations were filtered (Dong et al., 2020). Only the candidate regions identified in BSE-seq and BSR-seq were considered. Then, the Chinese Spring reference genome (RefSeq 2.1) was used to obtain the candidate gene, gene sequence, and gene annotation. Homologous analysis and gene expression patterns were evaluated on WheatOmics 1.0 (http://202.194.139.32/). Arabidopsis (TAIR10) and rice (IRGSP-1.0) genomes were used for comparative genomics analyses. A phylogenetic tree was constructed using MEGA11, and Geneious was employed to assemble high-quality reads.

### Differentially expressed gene analysis

Grains of *AK-3537* and AK58 during the grain filling stage (10 DPA) were collected. RNA extraction, library construction, and RNA sequencing were performed as described previously. Differentially expressed gene (DEG) analysis, Gene Ontology (GO) annotation, and Kyoto Encyclopedia of Genes and Genomes (KEGG) analysis were adopted to confirm the putative biological functions and biochemical pathways of DEGs (*AK-3537* and AK58) on the OmicsShare Tools (https://www.omicshare.com/).

### Validation of KASP markers and quantitative reverse transcription polymerase chain reaction

DNA extraction was performed as previously described, and KASP markers were used for genotyping in parents and the F_2_ population. The experimental method for KASP markers was referenced from previous reports (Li et al., 2020). The quantitative reverse transcription polymerase chain reaction (qRT-PCR) experiment was conducted to detect the expression levels of key genes involved in the wheat starch synthesis pathway, including *GBSSI*, *GBSSII*, *SBEI, SBEII, SSI, SSII, SSIIIa, SSIV, TaAGPL1, TaAGPS1a, TaBEI, TaBEIIa, TaBEIIb*, and *TaBEIII*. The experimental method for qRT-PCR was referenced from previous reports (Li et al., 2022). *Actin* was selected as the reference gene (Wang et al., 2014), and the relative quantification formula (2^−ΔΔ^Ct) ± standard error of the mean (SEM) was used to evaluate quantitative variation further. Three biological replicates were tested for each sample. All primers used in this study are listed in **Supplementary Table 1**.

## Results

### Characterization of the Dek mutant phenotype

**Figure 1:**
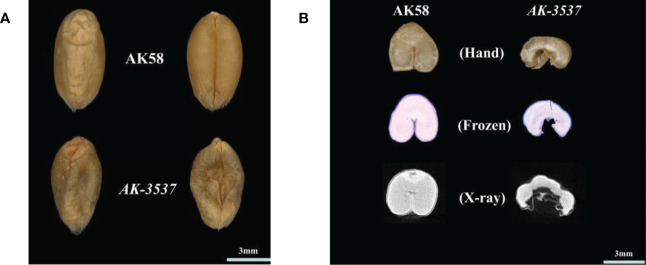
Wheat grain Dek phenotype and the internal structure of AK58 and *AK-3537*. **(A)** Dek phenotype of *AK-3537* mature grain compared to AK58 normal grain. **(B)** The internal structure of AK58 and *AK-3537* mature grains was observed by free-hand section (Hand), frozen section (Frozen), and x-ray tomography (X-ray).

In a screening of wheat grain, we identified a Dek mutant, *AK-3537*, with grain shrunken from an ethyl methyl sulfide (EMS) mutant library in the AK58 background (**Figure 1A**). The grains of the wild-type AK58 were plump and normal (AK58 type), whereas *AK-3537* showed a 100% grain Dek phenotype rate across all environments (*AK-3537* type) (**Figure 1**). To further establish the genetic basis of this phenotype, a cross was performed between *AK-3537* and AK58. All F_1_ plants exhibited a normal grain phenotype, suggesting the presence of a recessive gene controlling the wheat grain Dek phenotype. To assess *AK-3537* mutation segregation, (*AK-3537* × AK58) F_1_ plants were self-pollinated and an F_2_ segregating population (130 individuals) was developed. The F_2_ population was segregated into two categories: 95 plants exhibited an AK58-type phenotype, and 35 plants showed an *AK-3537*-type phenotype (**Table 1**). The results were in agreement with a 3:1 segregation ratio [χ^2^ = 0.26 < χ^2^ (0.05, 1) = 3.84, *p* = 0.61], suggesting that a single gene regulates grain Dek.

**Table 1:** Grain phenotype statistics of AK58 and *AK-3537* parents and F_2_ population.

	Name	Phenotype		Rate
Parents		AK58 type	*AK-3537* type	
	AK58	10		100%
	*AK-3537*		10	100%
F_2_ populatoin	*AK-3537* × AK58	95	35	3:01

**Figure 2:**
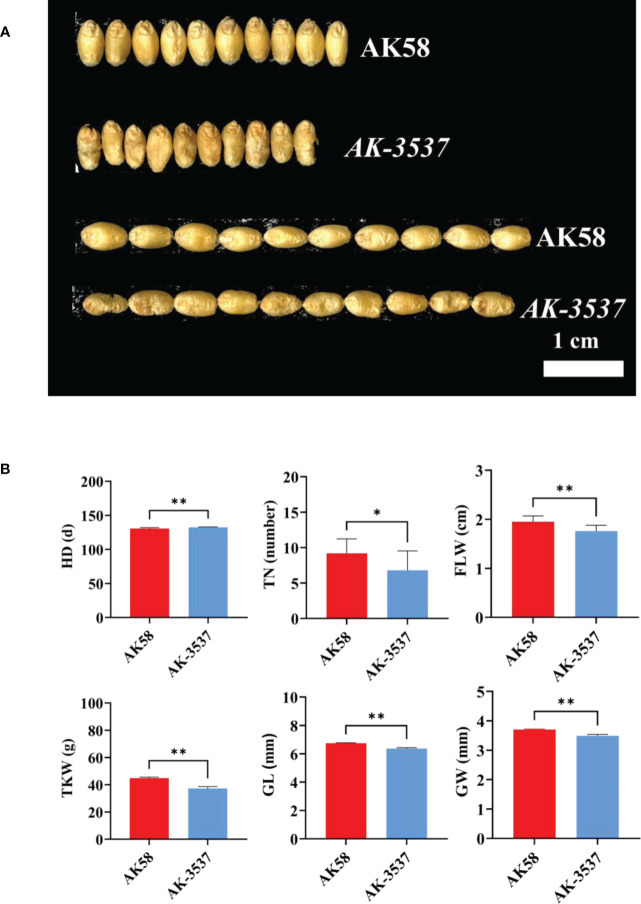
Comparison of grain and field phenotypes between AK58 and *AK-3537*. **(A)** Comparison of length and width phenotypes of *AK-3537* defective kernel and AK58 normal grain. **(B)** Statistical analysis of field phenotypes and grain phenotypes of AK58 and *AK-3537* including tiller number (TN), heading date (HD), flag leaf width (FLW), thousand kernel weight (TKW), grain length (GL), and grain width (GW). “*” means *p* < 0.05 and “**” means *p* < 0.01.

Compared with that in AK58, a large hollow area was observed in the endosperm of *AK-3537* by frozen and free-hand section, and the hollow phenotype was also observed in the Dek grain of *AK-3537* by X-ray 3D tomography (**Figure 1B**). The results showed that *AK-3537* grain filling was significantly affected. Finally, we evaluated the agronomic phenotypes of AK58 and *AK-3537* in the field. Compared with AK58, FLW, TN, TKW, GL, and GW were all significantly decreased in *AK-3537*, whereas HD increased considerably (**Figures 2A, B**).

### Dek gene mapping by BSE-seq and BSR-seq

For Dek gene mapping, two pooled samples, each comprising 30 normal and Dek phenotype F_2_ segregants, were constructed. Approximately 20 Gb of sequence data for the DNA pool and approximately 6 Gb for the RNA pool were generated and compared with the Chinese Spring reference genome (RefSeq V2.1) and AK58 genome. Then, we used the ΔSNP-index algorithm (Abe et al., 2012) for Dek gene mapping. ΔSNP-index higher than 0.7 in BSE-seq and BSR-seq Dek gene candidate region (DCR) was defined conservatively as the union of BSA-seq and BSR-seq credible intervals for candidate gene identification. In the AK58 genome, two DCRs, DCR1 and DCR2, were mapped on chromosome 7A by BSE-seq and BSR-seq (**Table 2**), among which DCR1 was located between 279.98 and 287.93 Mb on Chr7A with two SVs (structural variation) in two genes, and DCR2 was located between the range of 565.34 and 568.58 Mb on Chr7A with two SVs in two genes (**Table 2**). In the Chinese Spring reference genome (RefSeq V2.1), five DCRs, namely, DCR3, DCR4, DCR5, DCR6, and DCR7, were identified on chromosomes 1B, 7A, and 7B. DCR6 and DCR2 are the same candidate region and contain the same variant genes (**Table 2**). Because AK58 is a wheat–rye 1B/1R translocation line material, the genetic background of the 1B chromosome is very different between the Chinese Spring reference genome (RefSeq V2.1) and the AK58 genome (Jia J et al., 2021). It has also been reported that wheat chromosome 7B underwent structural rearrangement (Chen et al., 2020). Therefore, we speculated that DCR1 and DCR2 located on chromosome 7A were candidate regions for the wheat grain Dek phenotype, which was predicted to be a moderate functional effect (e.g., missense mutation).

**Table 2:** The wheat grain Dek candidate region in the AK58 genome and the Chinese Spring reference genome (RefSeq 2.1).

Region	Type	Chr	Pos	Gene	ΔSNP-index	Variant	Annotation	Genome
DCR1	BSR-seq	7A	279.98	*TraesCS7A03G0625900*	0.733	c.1049G>A	3-hydroxy-3-methylglutaryl-CoA synthase	AK58
	BSE-seq	7A	287.92	*TraesCS7A03G0631200*	0.733	c.1766C>T	Kinesin, motor region domain containing protein	
DCR2	BSR-seq	7A	565.34	*TraesCS7A03G0922700*	0.789	c.163G>A	FBD-associated F-box protein	
	BSE-seq	7A	568.58	*TraesCS7A03G0929200*	0.789	c.535G>A	S-adenosyl-L-methionine-dependent methyltransferases superfamily protein	
DCR3	BSE-seq	1B	0.88	*TraesCS1B03G0001300*	0.714	c.271G>A	Disease resistance protein (NBS-LRR class) family	Chinese spring
	BSE-seq	1B	1.80	*TraesCS1B03G0005100*	0.763	c.1553G>A	NBS-LRR-like resistance protein	
	BSE-seq	1B	4.14	*TraesCS1B03G0014000*	0.833	c.689G>A	Receptor protein kinase	
	BSE-seq	1B			0.7	c.723G>A	Receptor protein kinase	
	BSR-seq	1B	27.65	*TraesCS1B03G0099800LC*	0.783	c.106C>T	Polycystic kidney disease protein 1-like 2	
DCR4	BSE-seq	1B	125.71	*TraesCS1B03G0289600*	0.857	c.712G>A	Agmatine coumaroyltransferase-1	
	BSE-seq	1B	127.61	*TraesCS1B03G0293800*	0.875	c.1373C>T	Kinase family protein	
	BSE-seq	1B	137.88	*TraesCS1B03G0305800*	0.727	c.950C>T	NAC domain protein	
	BSE-seq	1B			0.727	c.949C>T	NAC domain protein	
	BSR-seq	1B	151.77	*TraesCS1B03G0332400*	0.739	c.569G>A	Translocase of chloroplast 159	
	BSR-seq	1B			0.739	c.-86G>A		
	BSR-seq	1B			0.739	c.568G>A		
	BSR-seq	1B			0.739	c.-87G>A		
	BSR-seq	1B			0.75	c.386C>T		
	BSR-seq	1B			0.75	c.-269C>T		
	BSR-seq	1B			0.75	c.376C>T		
	BSR-seq	1B			0.75	c.-279C>T		
DCR5	BSR-seq	1B	207.08	*TraesCS1B03G0399700LC*	0.812	c.209G>A	Transposon protein	
	BSE-seq	1B	218.79	*TraesCS1B03G0407700LC*	0.75	c.403G>A	Gag-pol polyprotein	
DCR6	BSR-seq	7A	559.38	*TraesCS7A03G0922700*	0.765	c.163G>A	FBD-associated F-box protein	
	BSE-seq	7A	562.69	*TraesCS7A03G0929200*	0.742	c.535G>A	S-adenosyl-L-methionine-dependent methyltransferases superfamily protein	
DCR7	BSE-seq	7B	761.91	*TraesCS7B03G1333300LC*	0.857	c.505C>T	Aspartate–tRNA(Asp/Asn) ligase	
	BSE-seq	7B			0.875	c.499G>A		
	BSR-seq	7B	763.24	*TraesCS7B03G1339800*	0.8	c.2653G>A	Rp1-like protein	

**Figure 3:**
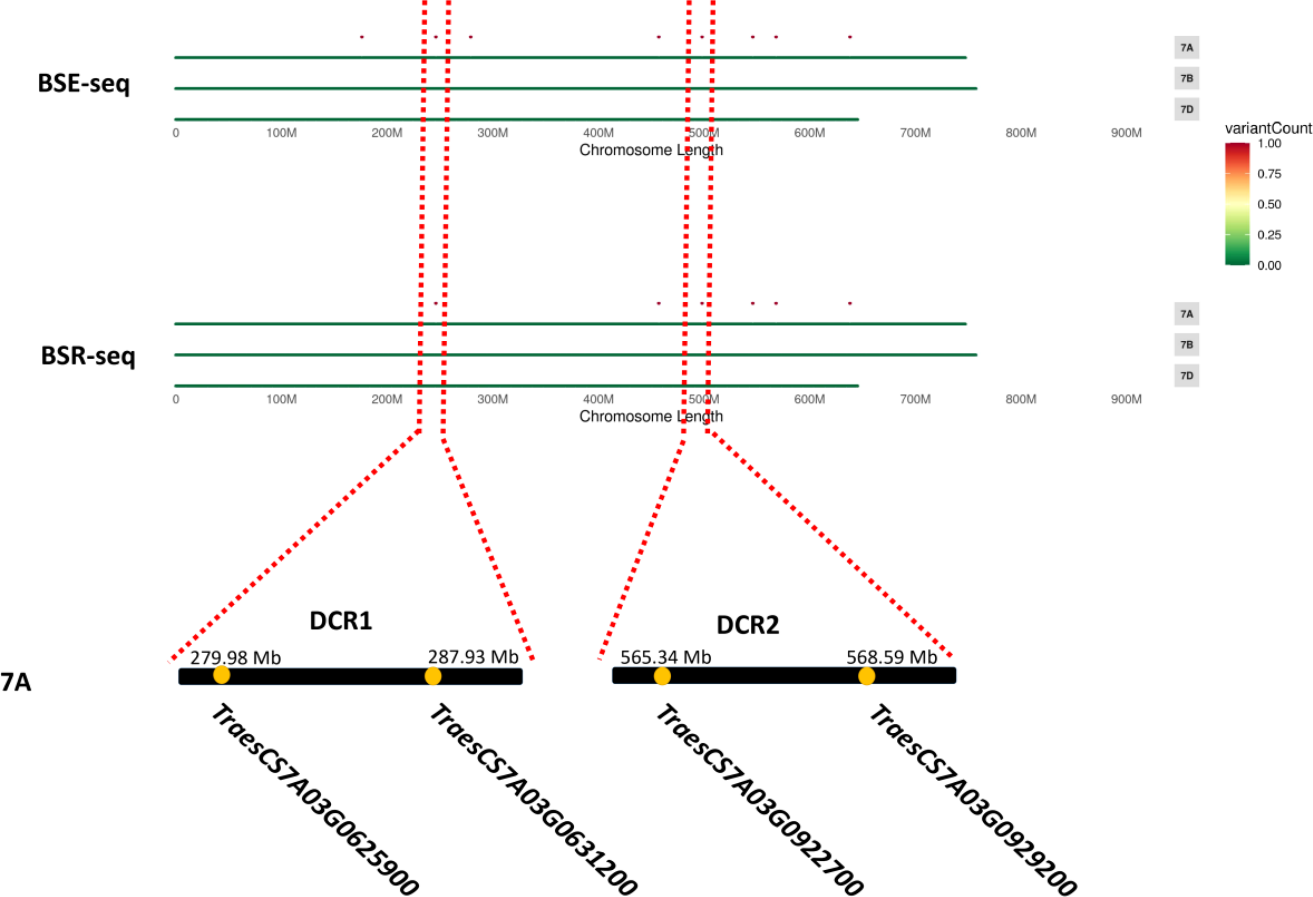
Most likely candidate region for wheat Dek detected via BSE-seq and BSR-seq. “.” means the SNPs associated with wheat grain Dek phenotype after the screening.

Since a single gene regulates the Dek phenotype, we annotated the genes in the two candidate regions based on the wheat Chinese Spring RefSeq v2.1 genome. In DCR1, a gene encoding 3-hydroxy-3-methylglutaryl-CoA synthase (*TraesCS7A03G0625900*) and a gene encoding a kinesin-like protein (*TraesCS7A03G0631200*) were annotated. In DCR2, an FBD-associated F-box protein (*TraesCS7A03G0922700*) and an S-adenosyl-L-methionine-dependent methyltransferase superfamily protein (*TraesCS7A03G0929200*) were annotated (**Figure 3**; **Table 2**). Expression patterns of the candidate genes in the WheatOmics 1.0 (http://202.194.139.32/) database revealed that all four candidate genes were expressed in seeds.

**Figure 4:**
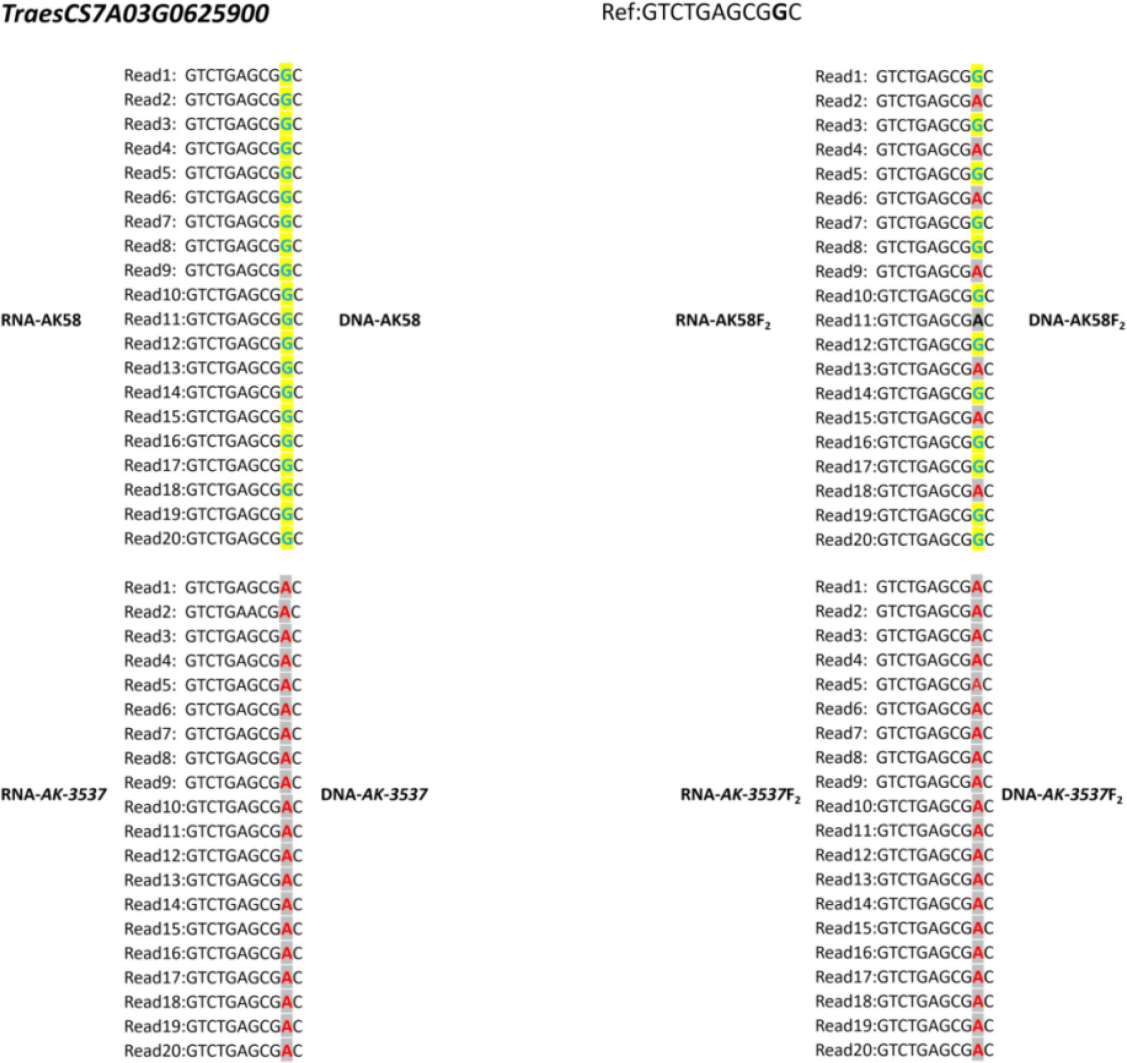
Candidate genes influencing wheat grain Dek phenotypes by the assembly of exome capture and RNA-seq sequencing data.

**Figure 5:**
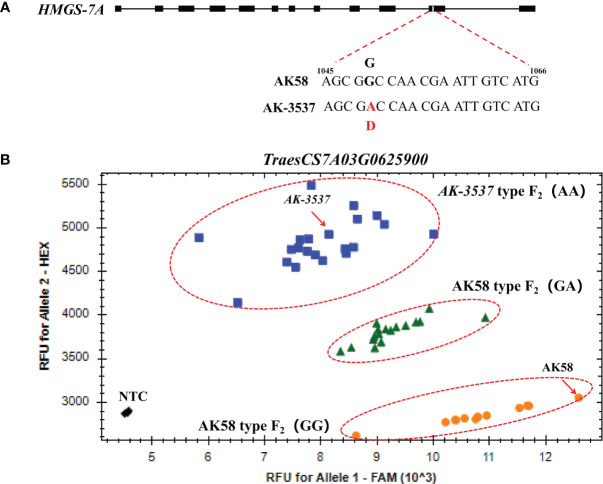
Mutation and marker verification of *HMGS-7A* gene. **(A)** Structure and mutation site of *HMGS-7A*. **(B)** *HMGS-7A^1049^* was validated by the KASP marker.

To further exclude SNP variations caused by sequence assembly errors, exome capture and RNA-seq data were used to assemble the sequences of the mutated genes in the DCR1 and DCR2 regions. The results showed that the SNP variations at *TraesCS7A03G0631200* and *TraesCS7A03G0922700* could be detected in both exome capture and RNA-seq data. However, the SNP of *TraesCS7A03G0929200* could only be detected in exome capture data, while the SNP of *TraesCS7A03G0625900* could only be detected in RNA-seq data (**Figure 4**; **Supplementary Figure 1**). To validate the segregation of these SNP variations in the F_2_ population, KASP markers were developed based on these four SNP variations in this study and validated in the F_2_ population. The results showed that only the SNP variation at position 1,049 of the *TraesCS7A03G0625900* coding region co-segregated with the grain normal and Dek phenotype in the F_2_ population (**Figure 5B**). A query of the Chinese Spring RefSeq v2.1 genome found that the coding sequence of the *TraesCS7A03G0625900* gene was 1,410 bp in length, and the full-length genome contained 12 exons and 11 introns. Sequencing analysis of the *TraesCS7A03G0625900* coding region in *AK-3537* revealed a G-to-A mutation at position 1,049, the 11th exon of the coding region, which resulted in an amino acid substitution from glycine (GGC, Gly, G) to aspartic acid (GAC, Asp, D) (**Figure 5A**). The target gene was tentatively termed *HMGS-7A*.

### Differentially expressed genes between AK58 and *AK-3537* grains

**Figure 6:**
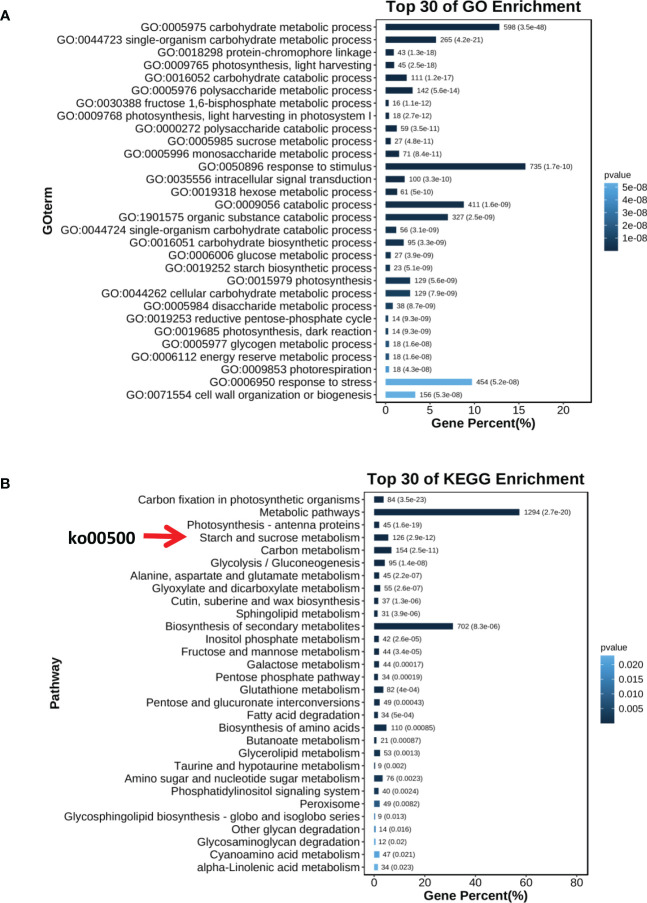
Differential expression analysis for *AK58* and *AK-3537* grain transcriptome. **(A)** GO analysis for DEGs. **(B)** KEGG analysis for DEGs.

**Figure 7:**
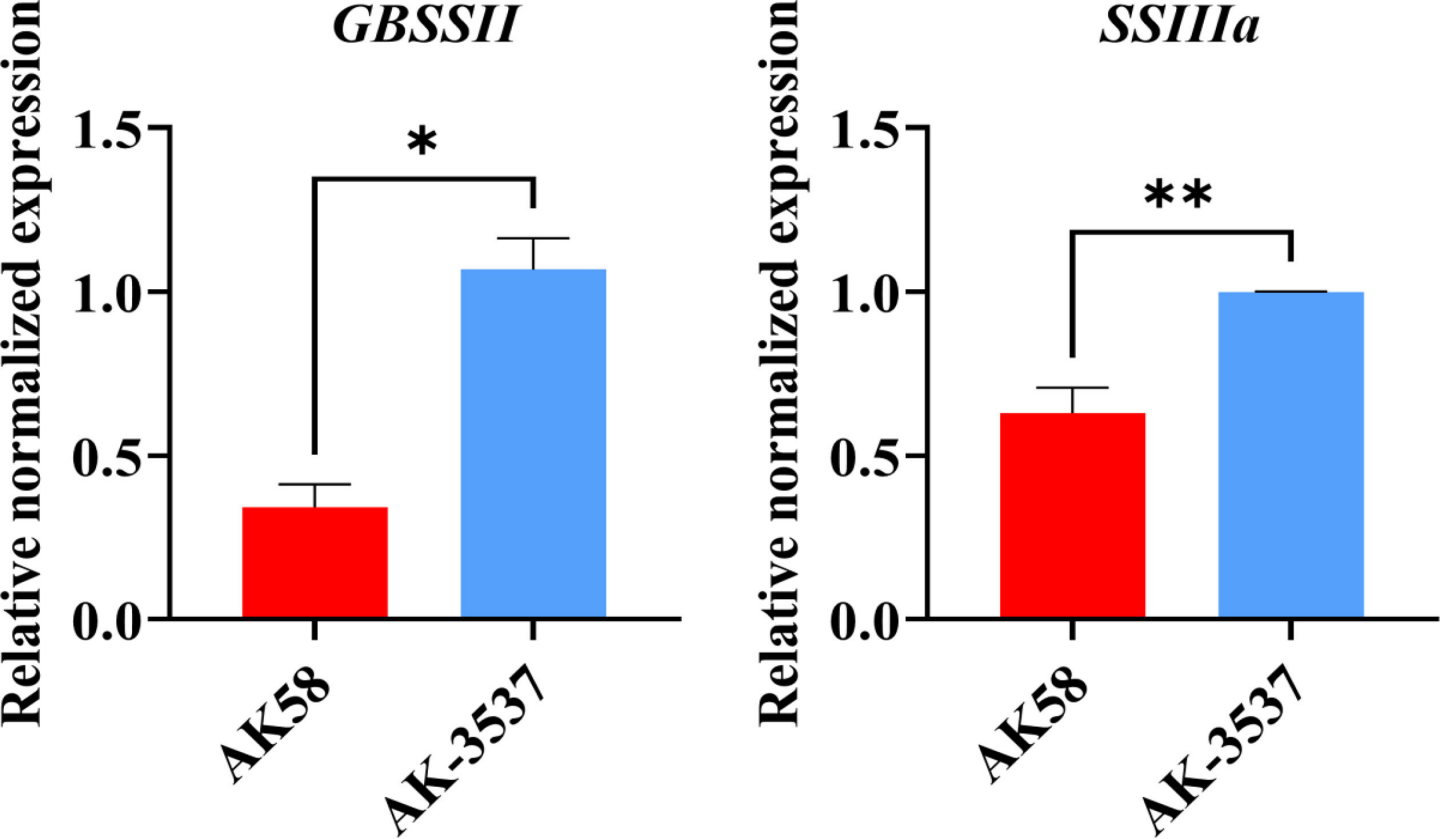
Statistical analysis of gene expression of starch synthetase *GBSSII* and *SSIIIa*.* means p < 0.05 and ** means p < 0.01.

In the transcriptome analysis of wheat grains of AK58 and *AK-3537*, a total of 12,655 DEGs were identified. Enrichment analysis of these DEGs in the wheat grain Dek regulatory pathway showed significant differences in gene expression levels between the mutant *AK-3537* and the wild-type AK58, with 6,618 genes downregulated and 6,037 genes upregulated. GO and KEGG analyses were further performed on the screened DEGs to understand the functions and pathways of these DEGs. GO analysis showed that these DEGs were mainly concentrated in processes involved in carbohydrate metabolism (GO:0005975 and GO:0044723) (**Figure 6A**). KEGG analysis showed significant enrichment (*p* ≤ 0.05) of energy metabolism and starch synthesis pathway (Ko00500) (**Figure 6B**). This indicates that, compared to AK58 grains, *AK-3537* grains have significant differences in wheat carbon metabolism, photosynthesis product synthesis, and starch synthesis pathways. These DEGs may cause the *AK-3537* grain Dek phenotype. In previous reports, starch synthase also regulated similar phenotypes (Morell et al., 2003; Li et al., 2011). Therefore, this study also found that the expression levels of *GBSSII* and *SSIIIa* in the Ko00500 pathway were significantly increased in *AK-3537* (**Figure 7**).

## Discussion

Wheat grain is the most important component of wheat yield and quality. In this study, we identified a candidate gene, *TraesCS7A03G0625900* (*HMGS-7A*), encoding a 3-hydroxy-3-methylglutaryl-CoA synthase (HMGS), in AK58 and *AK-3537* by BSE-seq and BSR-seq, responsible for the Dek phenotype in wheat grain. The G-to-A mutation at position 1,049 of the *HMGS-7A* coding region leads to amino acid change from glycine (Gly) to aspartic acid (Asp). In previous studies, several genes related to grain size and weight have been mapped and cloned on wheat chromosome 7A, including *TaTPP-7A* (7A, 135.0 Mb; Liu et al., 2023), *TaGASR7* (7A, 176.0 Mb; Zhang et al., 2015), *TaTGW-7A* (7A, 211.6 Mb; Hu et al., 2016), *TaGW8* (7A, 257.3 Mb; Yan et al., 2019), and *TaIAA21* (7A, 488.5 Mb; Jia M et al., 2021). The *TraesCS7A03G0625900* (*HMGS-7A*) gene identified in this study does not overlap with these reported grain morphology genes. Therefore, it is speculated to be a novel gene controlling wheat grain Dek phenotype.

**Figure 8:**
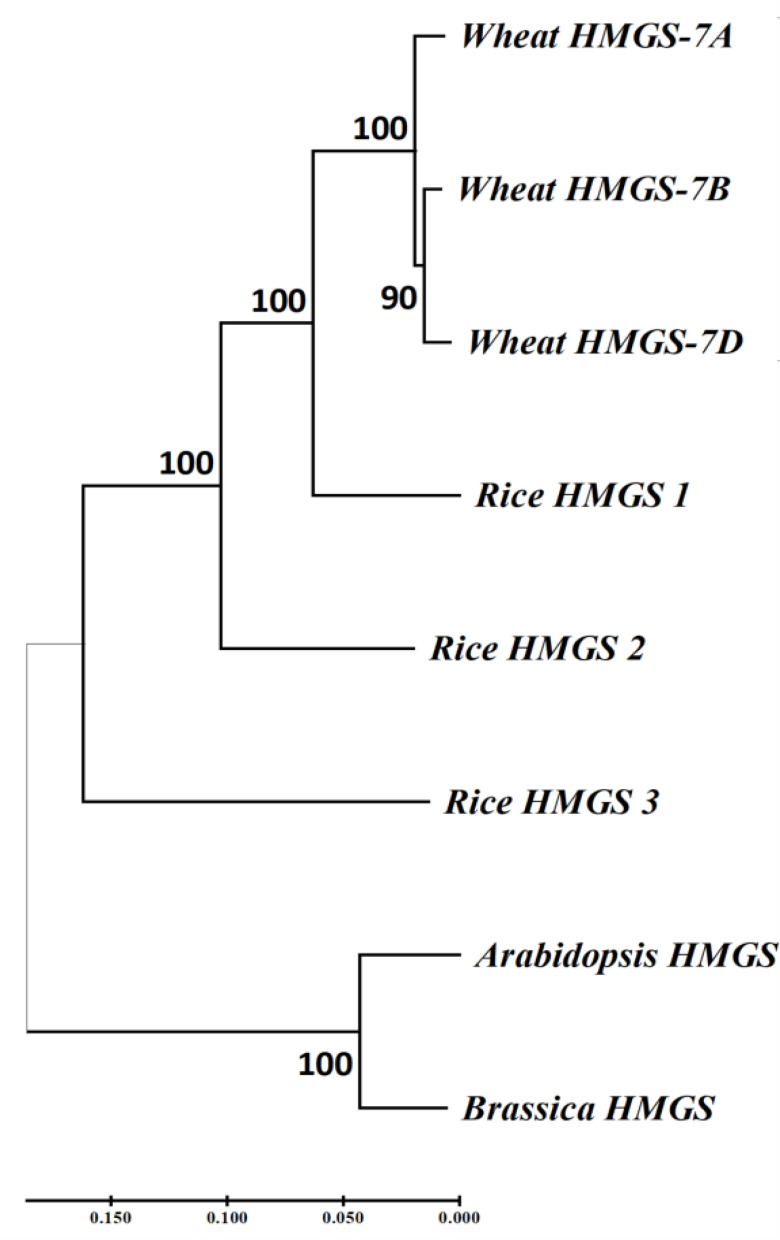
Phylogenetic analysis of *HMGS* homologous genes in wheat and *HMGS* in other plants.

The coding sequences of *TraesCS7A03G0625900* (*HMGS-7A*), and its two homologous genes, *TraesCS7B03G0451200* (*HMGS-7B*) and *TraesCS7D02G269600* (*HMGS-7D*), were obtained from Ensembl plants (https://plants.ensembl.org/index.html) and used to construct a phylogenetic tree with *HMGS* coding sequences from rice, Arabidopsis, and Brassica (**Figure 8**). The results showed that the *HMGS* in wheat is more closely related to that in rice. The importance of *HMGS* in Arabidopsis has been demonstrated in steroid biosynthesis, pollen fertility, and seed weight (Ishiguro et al., 2010; Bhangu-Uhlmann, 2011; Lange et al., 2015). HMGS is the second key enzyme in the mevalonate (MVA) pathway, significantly affecting plant sterol biosynthesis (Wang et al., 2012; Liao et al., 2014a; Lange et al., 2015; Pérez et al., 2022). Brassinosteroids (BR) is one of the main types of sterols (Zhang et al., 2020), which plays a vital role in the grain-filling process of plants. Transferring the gene encoding C-22 hydroxylase, an enzyme involved in sterol biosynthesis, into rice significantly increased the BR hormone content and the TKW, and the increase in TKW resulted from BR stimulating the transport of photosynthates in rice (Wu et al., 2008). Knockout of *TaD11-2A* results in dwarfism, a significant decrease in endogenous BR content, and smaller grains in wheat (Xu et al., 2022). *TaBRI1* is the BR receptor gene in wheat, and *TaBRI1* knockout mutants were insensitive to exogenous BR and significantly reduced TKW (Fang et al., 2020). *GW5* is a positive regulator of BR signaling, expressed in various rice organs, considerably affecting the width and weight of rice grains, and is a feasible target for increasing grain yield in rice and other cereal crops through gene editing (Liu et al., 2017). *SMG3* and *DGS1* regulate the size and weight of rice grains through the BR signaling pathway. Loss of *SMG3* or *DGS1* function results in smaller grains, while overexpression of *SMG3* or *DGS1* leads to longer grains (Li et al., 2023).

The sterol content in plant grains may also affect the starch synthesis pathway. A study of *sbeIIb* mutants in rice showed that many starch synthesis enzyme genes were upregulated, except for genes encoding granule-bound starch synthase, branching enzyme, pullulanase, and starch phosphorylase, which were downregulated. This increased amylose and resistant starch content, in addition to an increase in many other substances such as sugar, fatty acids, amino acids, and plant sterols in the endosperm (Baysal et al., 2020), and the wheat mutant *SM482gs*, with increased grain size, TKW, and protein content with BR biosynthesis and signal transduction, were significantly upregulated, but *AGP-S1*, *AGP-L2*, *SSI*, *SSIIa*, *SSIIIa*, *SBEIIa*, *SBEIIb*, and *GBSSIa* show the lower expression on *SM482gs* (Zhong et al., 2021), which indicated that plant sterols might be involved in the synthesis of amylose in plant grains. In rice, overexpression of *HMGS* significantly increased fatty acids, abscisic acid, gibberellins, and lutein in transgenic rice (Pérez et al., 2022), while overexpression of *HMGS* in mustard significantly increased grain weight (Liao et al., 2014b). In barley, mutations in the starch synthase genes *SSIIa* and *SSIIIa* result in grain phenotypes similar to those observed in this study with *AK-3537* (Li et al., 2011). Therefore, in this study, we detected the expression levels of key genes involved in starch synthesis in seeds and found that the key gene *SSIIIa*, which regulates the content of amylose and amylopectin in plants, was highly expressed in *AK-3537*, indicating that the functional changes of *HMGS-7A* may affect the expression of key enzyme genes involved in wheat starch synthesis. In the future, we will further analyze *HMGS-7A* and verify the role of *HMGS-7A* in wheat grain filling.

## Data availability statement

The datasets presented in this study can be found in online repositories. The names of the repository/repositories and accession number(s) can be found in the article/**Supplementary Material**.

## Author contributions

JW and HT designed research; HT performed experiments; HT and MC analyzed the data, ML, ZY, and ZP prepared the plant materials; HT, XG, HD, QC, and JW wrote and revised the paper; JW supervised the project. All authors contributed to the article and approved the submitted version.

## Supplementary material

Supplementary Figure 1 Candidate genes influencing wheat grain Dek phenotypes by assembly of exon capture and RNA-seq sequencing data.
