# Supplementary figures and images for: Identification of candidate gene for the defective kernel phenotype using bulked segregant RNA and exome capture sequencing methods in wheat
Source: Front Plant Sci. 2023 Jun 5;14:1173861. doi: 10.3389/fpls.2023.1173861 (PMC10277647; doi:10.3389/fpls.2023.1173861)

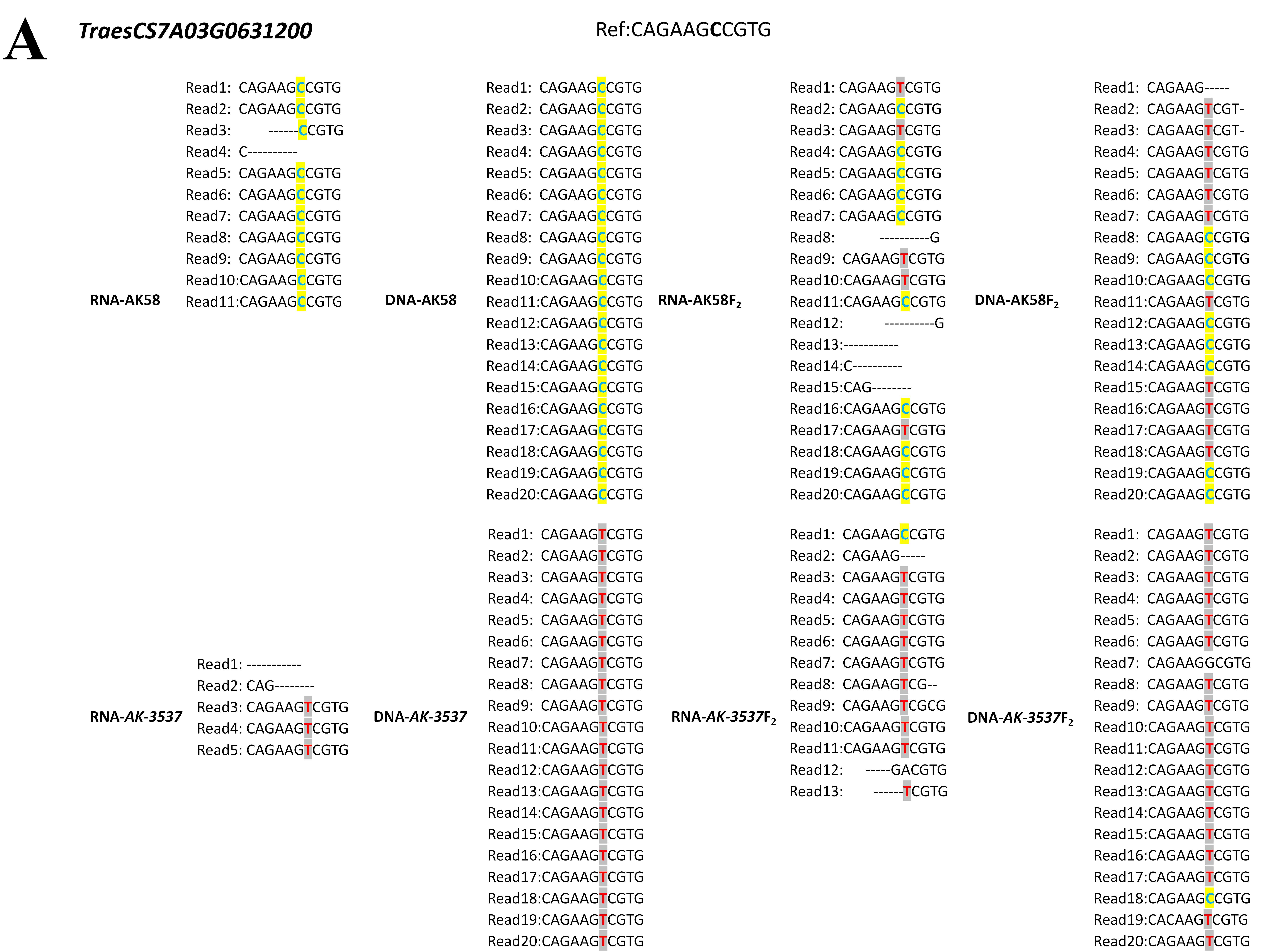

Supplement: Supplementary Figure 1 — Candidate genes influencing wheat grain Dek phenotypes by assembly of exon capture and RNA-seq sequencing data. [file Image_1.jpg]

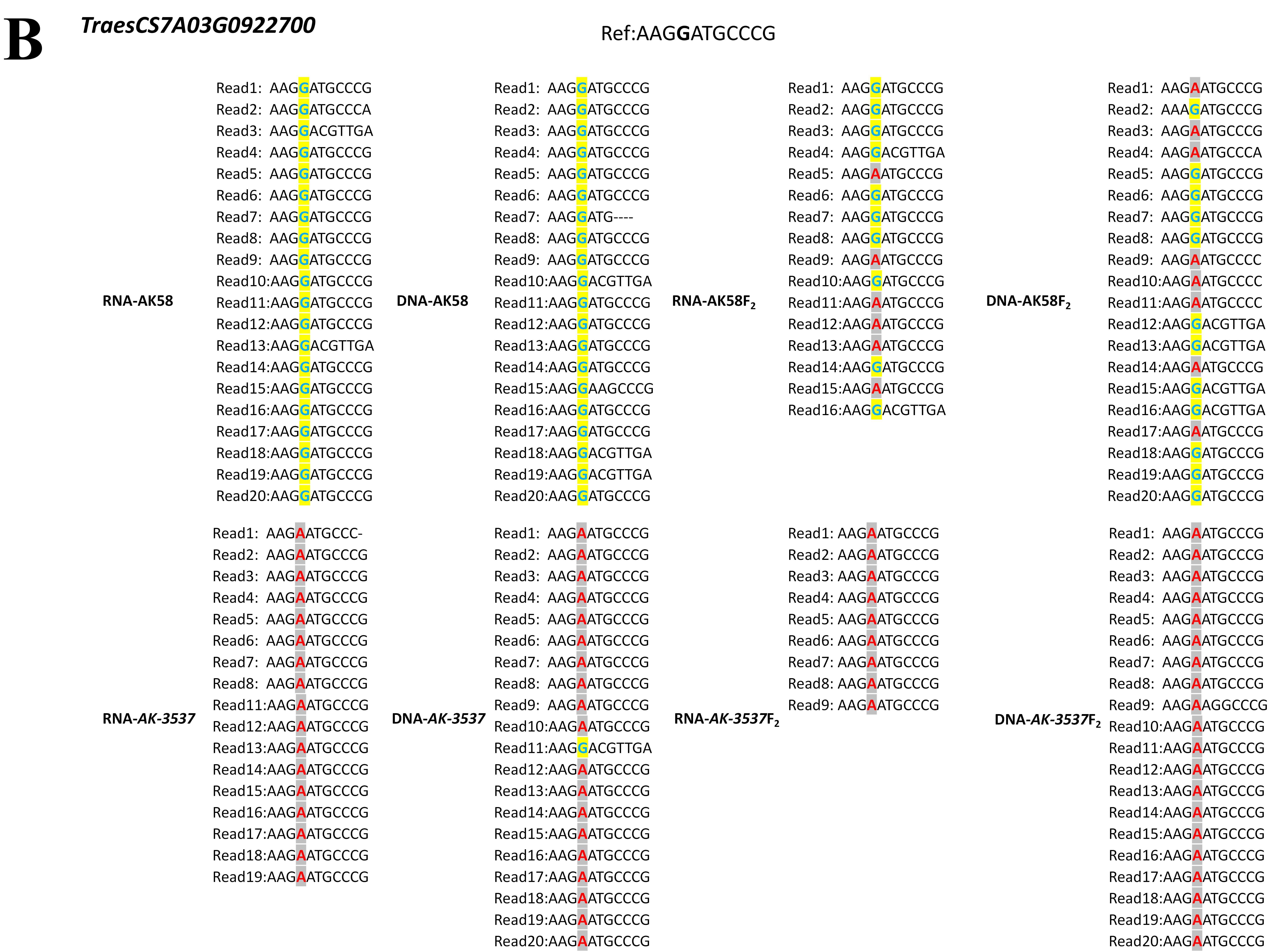

Supplement: Supplementary file 2 [file Image_2.jpg]

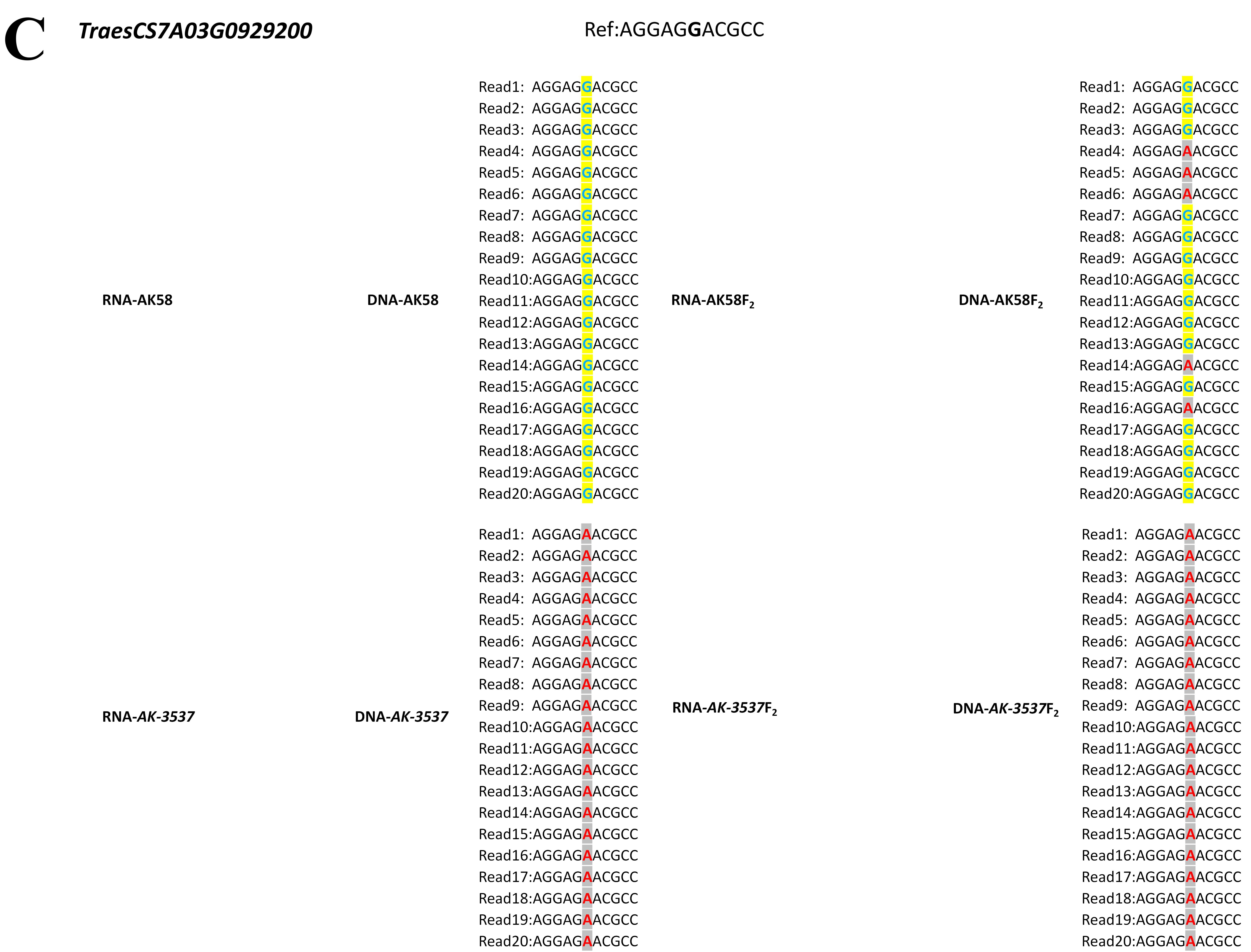

Supplement: Supplementary file 3 [file Image_3.jpg]
