# Supplementary material for: Identification of candidate gene for the defective kernel phenotype using bulked segregant RNA and exome capture sequencing methods in wheat
Source: Front Plant Sci. 2023 Jun 5;14:1173861. doi: 10.3389/fpls.2023.1173861 (PMC10277647; doi:10.3389/fpls.2023.1173861)
Supplement: Supplementary file 4 [file Table_1.docx]

**Supplementary Table 1.** Primers to be used in the study

| Name | Type | NCBI accession | Forward primer (5'-3') | Reverse primer (5'-3') | Reference |
| --- | --- | --- | --- | --- | --- |
| *Actin* | qPCR | DN551593 | GGAAAAGTGCAGAGAGACACG | TACAGTGTCTGGATCGGTGGT | Wang et al.,2014 |
| *GBSSI* | qPCR | AY050174 | GACACTATCGTGGAAGGCAAG | TTGACCATCTCATGGTACGC | Wang et al.,2014 |
| *GBSSII* | qPCR | AF109395 | CACAGAATGCCAGAGGCATAG | GAACAGATGGGAATCACTCCA | Wang et al.,2014 |
| *SBEI* | qPCR | AF286317 | ATGTTTGGTGGACATGGAAGA | ACGCGATAGTAAGCCACACAA | Wang et al.,2014 |
| *SBEII* | qPCR | Y11282 | GGGTTTAGGTGGTGAAGGCTA | AAATCTACGGCGGCATTTATC | Wang et al.,2014 |
| *SSI* | qPCR | AJ292521 | GCAAAAGGAGAGGAGGGTACA | ACGTATGGTCTTTCGTCATGC | Wang et al.,2014 |
| *SSII* | qPCR | AB201445 | GCTACACCAACTTCTCCCTG | GATGATCTCCACGCCCTTCT | Wang et al.,2014 |
| *SSIIIa* | qPCR | AF258608 | GACATGTGGTTTTGCTTGGTT | AGCCAGCGTATATCAGGTGAG | Kang et al., 2013 |
| *SSIV* | qPCR | AY044844 | CGACGAGCAGGGCCTAAGCA | AGCCGAGGTGTCCCAGCTGAA | Kang et al., 2013 |
| *TaAGPL1* | qPCR | Z21969 | GCCCCTGTTGGAGAGAGCCG | TAGCAGGGTCGTCGATGGCG | Kang et al., 2013 |
| *TaAGPS1a* | qPCR | X66080 | CCTTCCAAGCGTGAACAA | TTCCGAGAACACTATCATCAAC | Kang et al., 2013 |
| *TaBEI* | qPCR | Y12320 | TGGGTCGATCGGGTTCCTGCAT | ACGTGGAGCGTCAGGCTTTCG | Kang et al., 2013 |
| *TaBEIIa* | qPCR | AF286319 | GCAAGTCCGGCGCAACCTGA | CTCGCGGTTTCTCCCCCACG | Kang et al., 2013 |
| *TaBEIIb* | qPCR | AY740401 | CGCCTTCCATCGACGGTCCC | TCCCGGTGGTGGCAGAATGC | Kang et al., 2013 |
| *TaBEIII* | qPCR | JQ346193 | TACGTTGACAAGGATGCGCT | CTAATCCGCCTTGGGTGGTT | Kang et al., 2013 |
| *HMGS-7A^1049^* | KASP |  | GAAGGTGACCAAGTTCATGCTAACTTCCATTTCTTATGCAGAGCGg | CACTGCCGTAAGAGAACATGAC |  |
|  |  |  | GAAGGTCGGAGTCAACGGATTAACTTCCATTTCTTATGCAGAGCGa |  |  |
